# The Prognostic Value of Biomarkers in Patients Diagnosed with Spontaneous Bacterial Peritonitis

**DOI:** 10.3390/jcm15010208

**Published:** 2025-12-27

**Authors:** Süleyman Kırık, Mehmet Göktuğ Efgan, Ejder Saylav Bora, Efe Kanter, Ecem Ermete Güler, Tutku Duman Şahan

**Affiliations:** 1 Department of Emergency Medicine, Faculty of Medicine, Izmir Katip Çelebi University, 35100 Izmir, Turkey; kiriksuleyman2107@outlook.com (S.K.); goktugefgan@gmail.com (M.G.E.); saylavbora@hotmail.com (E.S.B.); efekanter@hotmail.com (E.K.); ecemermete@hotmail.com (E.E.G.); 2 Department of Emergency Medicine, Izmir Çeşme Alper Çizgenakat State Hospital, 35950 Izmir, Turkey

**Keywords:** spontaneous bacterial peritonitis, BUN/albumin ratio, RDW/albumin ratio, in-hospital mortality

## Abstract

**Background**: Spontaneous bacterial peritonitis (SBP) is a life-threatening complication of cirrhosis associated with high morbidity and mortality. Early risk stratification remains challenging due to the invasive nature of ascitic polymorphonuclear cell counting and the limited sensitivity of conventional markers. Novel composite biomarkers such as the blood urea nitrogen/albumin ratio (BAR), red cell distribution width/albumin ratio (RAR), and neutrophil-to-lymphocyte ratio (NLR) may offer practical prognostic value. This study aimed to evaluate the predictive power of these biomarkers for in-hospital mortality in patients diagnosed with SBP in the emergency department. **Methods**: This retrospective, observational, cohort study included adult patients diagnosed with SBP between March 2022 and October 2024. SBP diagnosis was confirmed by ascitic fluid leukocyte count > 250/mm^3^. Demographic data, laboratory parameters, and clinical outcomes were obtained from hospital records. RAR and BAR ratios were calculated at admission. Outcomes were analyzed according to ward/intensive care unit (ICU) admission and in-hospital mortality. Receiver operating characteristic (ROC) analysis and logistic regression were performed to assess prognostic performance. **Results**: A total of 112 patients were included (mean age 63.44 ± 13.16 years, 56.3% male). In-hospital mortality was 43.8%. Both BAR and RAR ratios were significantly higher in non-survivors (*p* = 0.016 and *p* = 0.001, respectively). BAR showed the highest prognostic performance (Area under the curve 0.761), with an optimal cutoff > 1.86 (sensitivity 64.44%, specificity 79.66%). RAR had an area under the curve (AUC) of 0.638, and NLR had an AUC of 0.658. **Conclusions**: Among the evaluated biomarkers, BAR emerged as the strongest predictor of in-hospital mortality in SBP, outperforming both RAR and NLR. Although albumin alone was not predictive, its use in composite ratios improved prognostic accuracy. These easily accessible biomarkers may support early risk stratification in emergency settings.

## 1. Introduction

Spontaneous bacterial peritonitis (SBP) is a serious and potentially fatal infection that develops in patients with cirrhosis and ascites. The clinical picture is characterized by a marked increase in renal failure and mortality risk, making early diagnosis and appropriate treatment strategies crucial [1]. Bacterial translocation, immune response disorders, and weakened intra-abdominal defence mechanisms play a key role in the pathogenesis of SBP. Recent research has focused on the use of various laboratory markers to accelerate diagnosis and improve prognosis [2]. In this context, composite indices such as albumin level and neutrophil/albumin ratio have emerged as valuable parameters for both diagnosis and predicting disease course [3].

Albumin is a critical protein not only in maintaining plasma oncotic pressure but also in defending against infections due to its anti-inflammatory and antioxidant properties. Low serum albumin levels have been associated with an increased risk of SBP development and poor prognosis [4]. However, ratios that evaluate albumin levels together with neutrophil counts have recently gained attention because they can simultaneously reflect inflammatory burden and nutritional status [5].

Red blood cell distribution width (RDW) is increasingly being investigated as a potential indicator of inflammation, oxidative stress, and nutritional deficiencies among hematological parameters; however, it has been addressed only to a limited extent in the existing literature in the context of SBP [6]. However, the possible role of RDW in inflammatory processes, when evaluated in conjunction with albumin and related ratios, may contribute to diagnosis and prognosis.

SBP represents a significant infection in individuals with cirrhosis, necessitating prompt diagnosis and intervention. Albumin and renal function markers, such as BUN and creatinine, serve as significant biomarkers in the diagnosis and prognosis of SBP. Recent studies have examined albumin-related ratios and novel biomarkers for their utility in diagnosing and monitoring treatment of SBP [7,8].

The range of peritoneal infections in cirrhotic patients includes not only SBP but also spontaneous fungal peritonitis (SFP) and secondary bacterial peritonitis, both of which can clinically and biochemically resemble SBP. Recent evidence indicates that spontaneous fungal peritonitis, while less prevalent, is linked to markedly increased mortality and delayed diagnosis owing to its nonspecific presentation and low culture sensitivity [9]. Distinguishing SBP from other peritoneal infections is crucial for prompt and precise treatment. Würstle et al. highlighted the diagnostic difficulties in differentiating SBP from secondary peritonitis and suggested laboratory and imaging criteria to assist clinicians in the earlier identification of secondary causes [10].

Conventional analysis of ascitic fluid is the fundamental diagnostic method; nonetheless, it is invasive, laborious, and occasionally yields inconclusive results. There is a growing exploration of non-invasive diagnostic alternatives. Cao et al. determined that ratios and serum biomarkers, such as C-reactive protein, neutrophil-to-lymphocyte ratio (NLR), and platelet indices, can reliably predict the development of spontaneous bacterial peritonitis (SBP) in cirrhotic patients, potentially diminishing the reliance on paracentesis [11]. These findings endorse the incorporation of systemic inflammatory markers into diagnostic algorithms, particularly in emergency contexts.

Recent years have witnessed an evolution in the microbiological profile of SBP, characterized by a rising prevalence of multidrug-resistant (MDR) organisms and Gram-positive pathogens. A Polish epidemiological study indicated significant regional variability in pathogen distribution, highlighting an increase in extended-spectrum beta-lactamase (ESBL)-producing Enterobacterales from 2017 to 2024 [12]. These findings highlight the necessity of ongoing microbiological surveillance and the requirement to reevaluate empirical antibiotic strategies in SBP.

Moreover, there is increasing acknowledgment of the gut microbiota’s crucial role in the pathogenesis of SBP. Dysbiosis in advanced chronic liver disease facilitates bacterial translocation and immune dysregulation, increasing the susceptibility of patients to infections such as spontaneous bacterial peritonitis [13]. The restoration of gut microbial equilibrium via targeted therapies may serve as a prospective preventive or adjunctive approach for managing SBP.

SBP is a complication that causes serious morbidity and mortality in patients with cirrhosis and requires rapid diagnosis and treatment. The classic diagnostic method, counting polymorphonuclear (PMN) cells in ascites fluid, is invasive and has a low culture positivity rate; therefore, studies are being conducted to develop new biomarkers and models for early and non-invasive diagnosis. Taken together, these findings highlight the complexity of SBP diagnosis and management, reflecting multifactorial interactions between microbial ecology, host immunity, and systemic inflammation. Therefore, identifying novel, non-invasive, and easily measurable biomarkers such as BAR, RAR, and NLR is of growing clinical relevance to support early diagnosis and prognosis in cirrhotic patients presenting with ascites.

## 2. Materials and Methods

### 2.1. Study Design

This retrospective, observational cohort study was conducted at the Emergency Department of a tertiary university hospital that serves as a referral center for patients with advanced liver disease. The study period extended from 1 March 2022 to 1 October 2024. The hospital receives both direct emergency admissions and transfers from secondary care facilities, providing a representative sample of patients with moderate-to-severe cirrhosis.

The study was designed to evaluate the prognostic utility of easily accessible biomarkers namely, the blood urea nitrogen-to-albumin ratio (BAR), red cell distribution width-to-albumin ratio (RAR), and neutrophil-to-lymphocyte ratio (NLR) for predicting in-hospital mortality in patients with spontaneous bacterial peritonitis (SBP). The study followed the Strengthening the Reporting of Observational Studies in Epidemiology (STROBE) guidelines for observational research.

### 2.2. Study Population

The population of this study consists of adults aged 18 years and older who visited the hospital’s emergency department between 1 March 2022 and 1 October 2024.

**Figure 1:**
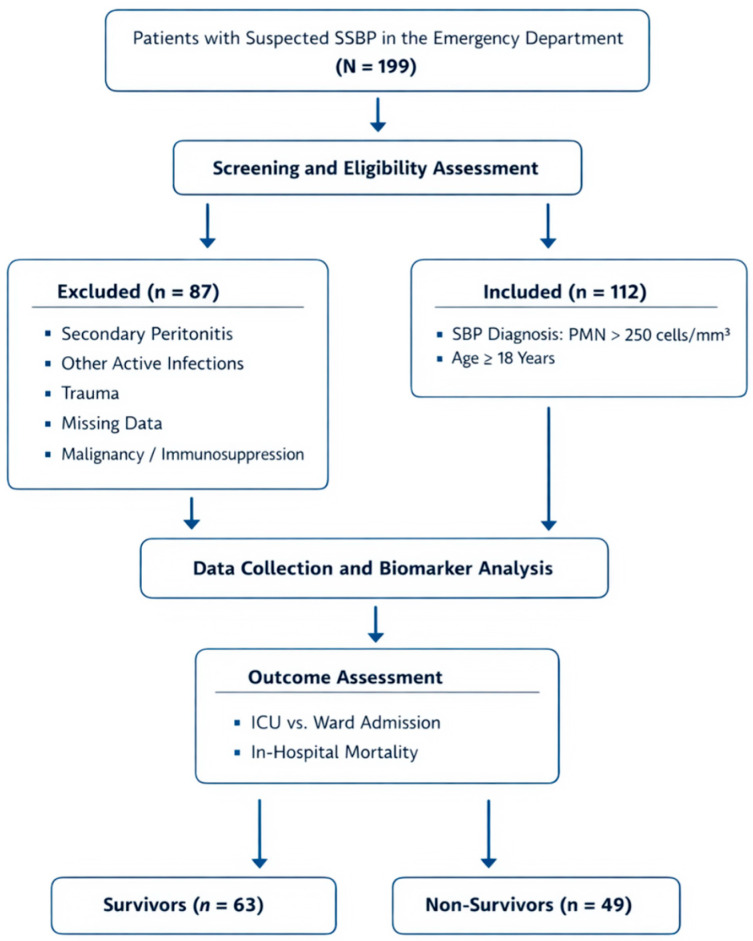
Study flowchart.

Inclusion criteria included: Confirmed spontaneous bacterial peritonitis, defined by an ascitic fluid polymorphonuclear (PMN) cell count > 250/mm^3^ obtained from a sterile paracentesis sample. Patients with other concurrent intra-abdominal pathological conditions (e.g., diverticulitis, acute cholecystitis), patients with concurrent infections at other anatomical sites (e.g., pneumonia, urinary tract infection, bloodstream infection), trauma patients, those with missing baseline laboratory data at the time of admission, patients whose outcome could not be followed up (those who were transferred or refused treatment), pregnant women and women who were breastfeeding, and patients with active malignancy or receiving immunosuppressive therapy were excluded from the study. These exclusion criteria were established to increase the homogeneity of the patient population and strengthen the internal validity of comparative analyses. During the study period, a total of 199 patients were screened for eligibility. Of these, 25 patients were excluded due to missing baseline laboratory data, 17 due to concurrent intra-abdominal infections, 13 due to transfer to another center, and 32 due to other predefined exclusion criteria. After applying all exclusion criteria, 112 patients were included in the final analysis. The study was conducted at a tertiary referral center with a high-volume emergency department that serves both primary presentations and referred patients with advanced liver disease from surrounding hospitals. As a result, the study population may include a higher proportion of clinically severe cases compared with community-based centers (Figure 1).

### 2.3. Data Collection

Basic clinical parameters such as demographic data (age, gender, etc.), laboratory parameters, and hospital admission status for patients included in the study were obtained retrospectively from the hospital information system. All eligible patients who met the inclusion criteria during the study period were consecutively enrolled to minimize selection bias. No sampling or case selection was performed beyond the predefined inclusion and exclusion criteria. Additionally, the RDW/albumin ratio (RAR) and BUN/albumin ratio (BAR) were calculated from the hemogram and biochemistry parameters obtained from laboratory tests performed at the time of admission. These data were analysed comparatively according to service/intensive care admission, discharge, and mortality factors in order to assess the predictability of patient prognosis.

### 2.4. Statistical Analysis

Statistical analyses were performed using IBM SPSS Statistics version 26.0 (IBM Corp., Armonk, NY, USA). Continuous variables were expressed as mean ± standard deviation (SD), and categorical variables as counts and percentages. Normality testing: The Shapiro–Wilk test was used to assess the normal distribution of continuous variables.

Group comparisons: Normally distributed variables were analyzed using the independent-samples *t*-test, and non-normally distributed variables using the Mann–Whitney U test. Categorical variables were compared using the chi-square (χ^2^) test or Fisher’s exact test where appropriate.

Prognostic performance: Receiver operating characteristic (ROC) curves were generated to evaluate the discriminative ability of BAR, RAR, and NLR for in-hospital mortality. The area under the curve (AUC) and 95% confidence intervals (CIs) were calculated. The optimal cutoff values were determined using the Youden index.

Regression analysis: Univariate and multivariate logistic regression models were used to identify independent predictors of in-hospital mortality. Variables with *p* < 0.10 in univariate analysis were included in the multivariate model.

Significance threshold: statistical significance was defined as *p* < 0.05 (two-tailed).

## 3. Results

The average age of patients was 63.44 ± 13.16. The patient group consisted of 56.3% men (63 individuals) and 43.8% women (49 individuals). Ward/ICU Admission: 56.7% of patients were admitted to the ward (59 patients), while 43.3% were admitted to the Intensive Care Unit (ICU) (45 patients). Discharge/Death: 56.3% of patients were discharged (63 patients), while 43.8% died (49 patients). The mean RDW/ALB was 0.76 ± 0.26. The mean BUN/ALB was 1.84 ± 1.32; the mean NLR value was 25.36 ± 67.52. (Table 1).

There is a statistically significant difference between the variables PLT, PCT, BUN, AST, T.BIL, D.BIL, CRP, outcome, and BUN/ALB and the emergency department outcome (*p* < 0.05) (Table 2). The mean platelet count and PCT value of patients admitted to the ICU were statistically significantly lower than those admitted to the ward. The mean BUN of patients admitted to the ICU was statistically significantly higher than that of patients admitted to the ward. The mean AST, mean total bilirubin, and mean direct bilirubin levels of patients admitted to the ICU were significantly higher than those of patients admitted to the ward. The mean CRP level of patients admitted to the ICU was significantly higher than that of patients admitted to the ward. The BUN/ALB ratio in patients admitted to the ICU is significantly higher than in those admitted to the ward. The mortality rate in patients admitted to the ICU (63.3%) is much higher than in those admitted to the ward (36.7%). No significant difference was found in NLR values in the context of ICU admission (Table 2).

The lymphocyte count, mean platelet count, and PCT value of patients who died were statistically significantly lower than those of patients who were discharged. The mean values of RDW, BUN, creatinine, AST, ALT, total bilirubin, direct bilirubin, and CRP in patients who died were statistically significantly higher than those in patients who were discharged (Table 3). While 68.9% of patients admitted to the ICU died, only 30.5% of patients admitted to the ward died. The RDW/ALB and BUN/ALB ratios in patients who died were significantly higher than in those discharged. Similarly, the NLR value was also found to be significantly higher (Table 3).

**Figure 2:**
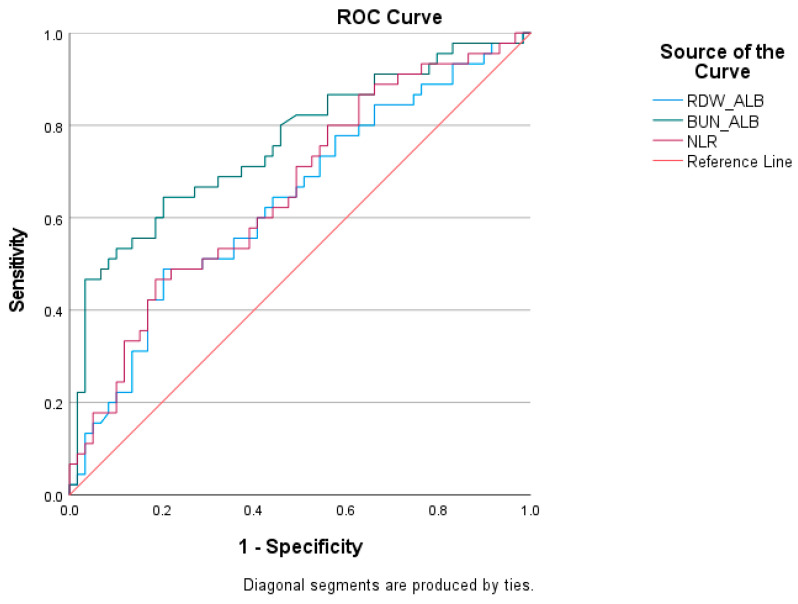
ROC curve of biomarkers RDW/ALB, BUN/ALB, NLR.

According to the table, the highest area under the curve (AUC) value belongs to BUN/ALB, while the lowest AUC value belongs to the RDW/ALB parameter. The optimal cutoff value for BUN/ALB was found to be >1.86, with a sensitivity of 64.44% and a specificity of 79.66%. The optimal cutoff value for RDW/ALB was found to be >0.83, with a sensitivity of 48.89% and a specificity of 79.66%. The optimal cutoff value for NLR was found to be >13.13, with a sensitivity of 53.30% and a specificity of 57.60% (Table 4), (Figure 2). The ROC derived cutoff values should be regarded as exploratory and require external validation before clinical application.

## 4. Discussion

In patients with cirrhosis, novel biomarkers are being progressively explored in conjunction with conventional clinical scores to forecast the onset and prognosis of SBP [14,15]. Recent years have witnessed the emergence of novel biomarkers in ascitic fluid and blood that demonstrate potential in forecasting both short- and long-term mortality risk associated with SBP [16]. In this study, RDW/ALB and BUN/ALB NLR biomarkers were investigated for mortality, and they were found to be early and usable biomarkers for predicting in-hospital mortality.

Similar to our findings, Long et al. emphasized that SBP remains a major cause of mortality in cirrhotic patients, with rates ranging between 15% and 40%, and highlighted the need for reliable, non-invasive prognostic tools that can be utilized promptly in the emergency department [1]. This underscores the relevance of composite biomarkers such as BAR and RAR in rapid clinical assessment.

Research indicates that renal impairment is a key determinant of prognosis in SBP. In a 13-year retrospective cohort study, Ubhi et al. demonstrated that markers of renal dysfunction at hospital admission were strongly associated with increased mortality in patients hospitalized with spontaneous bacterial peritonitis. [17]. In this study, it was similarly observed to be a strong independent predictor of in-hospital mortality. This renal dysfunction may be thought to increase the toxic load accumulated in cirrhotic patients in particular, thereby triggering the emergence of central findings. Consistent with this, it was noted that “acute kidney injury is the primary predictor of mortality in patients with SBP,” further supporting our conclusion that BAR—reflecting both renal and nutritional status—serves as an integrated indicator of disease severity [1].

Many studies describe that elevated NLR is a straightforward, non-invasive indicator linked to heightened risk and unfavorable prognosis in SBP [15,18,19,20]. In a prospective observational diagnostic accuracy study, they described that NLR and CRP for early detection of SBP are significantly useful. Moreover NLR ≥ 6.8 detected SBP with 82% sensitivity and 68% specificity for detecting SBP [13]. In our study sensitivity and specificity are 62.20% and 51.59%, respectively, for the NLR > 13.13 for predicting mortality. This is similar to the study of Efgan et al. who found similar sensitivity and specificity in 275 patients in predicting mortality. Despite the specificity and sensitivity results being similar to this study, the cut-off value differs significantly. This difference may be attributed to variations in patient numbers and inflammatory burden. In a study by IIiaz et al., the 30-day mortality rate for SBP was 26% [21]. Moreover, in this study 43.8% of patients died. The in-hospital mortality rate observed in the present study (43.8%) is higher than that reported in many SBP cohorts, where mortality rates typically range between 20% and 30%. This value is closer to the upper limit described by Long et al., who noted that mortality “per episode of SBP ranges from 15% to 40%,” particularly in severe cases or delayed presentations [1]. This difference may be explained by the characteristics of our study population, which consisted of patients diagnosed at emergency department presentation in a tertiary referral center. A substantial proportion of patients required intensive care unit admission, reflecting advanced disease severity and acute clinical deterioration at the time of diagnosis. In addition, delayed presentation, severe systemic inflammation, and the presence of acute complications at admission may have contributed to the higher mortality observed in this cohort. Compared to the mortality rate, the inflammatory burden reflects the difference in NLR cut-off.

The RAR is emerging as a straightforward, economical biomarker for forecasting prognosis and mortality in diverse critical conditions, including SBP in cirrhotic patients. Recent studies have investigated its diagnostic and prognostic significance, particularly in sepsis, hepatic disorders, and associated infections [22,23]. Although direct research on RAR in SBP is scarce, RDW alone has a negative correlation with survival in SBP patients, and low albumin is associated with poorer outcomes [24,25]. Similarly, in this study, according to the hospital outcomes, RDW has the power to assess hospital mortality. On the other hand, RAR also has a strong prognostic value in predicting mortality even though the albumin value alone is not meaningful. This shows us that RAR encompasses both inflammatory and hepatic/nutritional status, rendering it a reliable marker for risk stratification in critical illness as well as SBP.

Different findings were reported by Naeimi Bafghi et al. who emphasized that MPV, another readily available CBC parameter, could serve as a “non-invasive, simple, and accessible laboratory predictor” for SBP, supporting the value of easily obtainable ratios in clinical prognosis [26]. This shows us that RAR encompasses both inflammatory and hepatic/nutritional status, rendering it a reliable marker for risk stratification in critical illness as well as SBP.

Elevated BAR indicates both heightened inflammatory burden and impaired nutritional/renal status, rendering it a sensitive indicator of disease severity in inflammatory conditions [27,28,29]. In cirrhosis and similarly in SBP, the rise in BUN is attributed to multiple factors, including renal dysfunction prevalent in advanced liver disease, gastrointestinal bleeding that elevates BUN levels, and hypoalbuminemia resulting from inadequate nutrition and inflammation. A high BAR indicates a cumulative burden of complications, serving as a comprehensive marker for mortality as well as disease severity risk [30,31]. In this study, BAR demonstrated stronger predictive power for in-hospital mortality than NLR and RAR. Although it outperformed both parameters when sensitivity and specificity were considered together, it was still found to be unable to serve as a stand-alone or absolute prognostic indicator. More findings were reported by Naeimi Bafghi et al. who emphasized that MPV, another readily available CBC parameter, could serve as a “non-invasive, simple, and accessible laboratory predictor” for SBP, supporting the value of easily obtainable ratios in clinical prognosis [26]. This shows us that RAR encompasses both inflammatory and hepatic/nutritional status, rendering it a reliable marker for risk stratification in critical illness as well as SBP. Moreover, in the emergency medicine literature, which highlights that clinical impression alone is insufficient for SBP identification, Chinnock et al. demonstrated that “physician clinical impression had a sensitivity of 76% and specificity of 34%,” concluding that diagnostic reliability improves when supported by laboratory analysis [32]. Hence, biomarkers such as BAR can augment traditional diagnostic pathways, especially when rapid paracentesis or culture data are unavailable in emergency settings.

This result indicates that it cannot be used as an absolute mortality indicator when evaluated alone. Furthermore, our study demonstrates that BUN values alone are not meaningful, and that mortality rates associated with changes in BUN values are the most important indicator of mortality in SBP and renal failure. Although several biomarkers demonstrated statistically significant associations with in-hospital mortality, the relatively limited sample size of the present study may have reduced the statistical power to detect smaller effect sizes. Therefore, nonsignificant findings should be interpreted with caution. Larger, multicenter studies with higher patient numbers are warranted to further validate these results. We hope that subsequent studies with larger patient numbers will support these findings.

Unlike the other studies and results mentioned above, our study found that albumin alone was not meaningful in determining in-hospital mortality, but that a proportional approach using values such as albumin and RDW yielded meaningful results. Taken together with the findings of Long et al. [1], Naeimi Bafghi et al. [26], and Chinnock et al. [32], our results reinforce the evolving understanding that combined, easily obtainable biomarkers can effectively complement classical ascitic analyses and clinical judgment in assessing SBP severity and prognosis.

## 5. Limitations

The retrospective, single-center design may restrict the generalizability of the results. Biomarker measurements were limited to baseline values obtained at admission, and longitudinal changes were not assessed. An important limitation of this study is the inability to incorporate established liver disease severity scores such as the Model for End-Stage Liver Disease (MELD) or Child–Pugh classification into the multivariable analysis. Due to the retrospective nature of the study and incomplete documentation in hospital records, these scores could not be reliably calculated for all patients. Therefore, the findings should be interpreted primarily in the context of emergency department-based laboratory parameters. Future large-scale, multicenter studies are warranted to formally evaluate BAR within multivariable prognostic frameworks Owing to the observational study design, causal inferences cannot be drawn.

## 6. Conclusions

In conclusion, in this study involving our patient numbers and isolated SBP cases, although albumin alone was not significant, the BUN/albumin ratio was found to be a stronger predictor of prognosis than the NLR and RDW ratios studied.

## Figures and Tables

**Table 1:** Descriptive statistics for patients.

	Statistics
**Age**	63.44 ± 13.16
**Gender**	
Female	49 (43.8)
Male	63 (56.3)
**WBC**	15.76 ± 9.19
**NEU**	14.29 ± 11.19
**LYM**	1.08 ± 0.74
**MONO**	0.97 ± 0.68
**HGB**	13.25 ± 16.32
**HCT**	31.9 ± 5.56
**PLT**	262.67 ± 175.95
**PCT**	0.28 ± 0.17
**RDW**	17.79 ± 3.13
**BUN**	43.32 ± 29.29
**CRE**	1.65 ± 1.18
**AST**	89.15 ± 137.04
**ALT**	49.42 ± 109.23
**ALB**	24.69 ± 5.77
**T. BIL**	3.58 ± 4.76
**D. BIL**	2.11 ± 3.46
**CRP**	147.24 ± 107.17
**Emergency Department Outcome**	
Ward	59 (56.7)
ICU	45 (43.3)
Discharge	63 (56.3)
Deceased	49 (43.8)
**RDW/ALB**	0.76 ± 0.26
**BUN/ALB**	1.84 ± 1.32
**NLR**	25.36 ± 67.52

**Table 2:** Comparison of parameters according to emergency department outcomes.

	Ward	ICU	Test Statistics	*p*
**Age**	64.85 ± 12.77	60.62 ± 13.53	1.569	0.117 ^†^
**Gender**			0.416	0.519 ^†^
Female	26 (60.5)	17 (39.5)		
Male	33 (54.1)	28 (45.9)		
**WBC**	15.76 ± 9.62	16.91 ± 8.96	0.932	0.352 ^†^
**NEU**	13.56 ± 9.05	16.58 ± 13.78	1.322	0.186 ^†^
**LYM**	1.03 ± 0.66	1.05 ± 0.68	0.059	0.953 ^†^
**MONO**	1 ± 0.72	0.99 ± 0.67	0.075	0.940 ^†^
**HGB**	10.56 ± 1.77	17.4 ± 25.24	1.372	0.170 ^†^
**HCT**	31.94 ± 4.98	32.06 ± 5.98	0.112	0.911 ^†^
**PLT**	297.97 ± 193.6	205.71 ± 135.62	2.611	**0.009** ^†^
**PCT**	0.3 ± 0.18	0.23 ± 0.15	2.161	**0.031** ^†^
**RDW**	17.74 ± 3.28	18.08 ± 3.09	0.515	0.606 ^†^
**BUN**	37.49 ± 24.91	52.96 ± 33.65	2.264	**0.024** ^†^
**CRE**	1.45 ± 0.8	2.01 ± 1.54	1.811	0.070 ^†^
**AST**	71.02 ± 93.59	122.84 ± 181.29	2.145	**0.032** ^†^
**ALT**	45.25 ± 83.57	60.89 ± 143.21	1.139	0.255 ^†^
**ALB**	24.81 ± 5.78	24.42 ± 5.53	0.687	0.492 ^†^
**T. BIL**	2.28 ± 2.62	5.72 ± 6.26	3.042	**0.002** ^†^
**D. BIL**	1.26 ± 1.79	3.47 ± 4.7	2.581	**0.010** ^†^
**CRP**	129.3 ± 88.79	181.23 ± 124.54	2.165	**0.030** ^†^
**Outcome**			15.092	**0.001** ^†^
Discharge	41 (74.5)	14 (25.5)		
Deceased	18 (36.7)	31 (63.3)		
**RDW/ALB**	0.76 ± 0.27	0.78 ± 0.26	0.246	0.806 ^†^
**BUN/ALB**	1.62 ± 1.35	2.24 ± 1.28	2.602	**0.009** ^†^
**NLR**	16.62 ± 1.77	40.06 ± 15.54	0.594	0.441

Given as *mean ± standard deviation.* ^†^: Mann–Whitney U test, statistically significant *p*-values are bolded.

**Table 3:** Comparison of parameters according to in-hospital outcomes.

	Discharge	Deceased	Test Statistics	*p*
**Age**	63.41 ± 12.34	63.47 ± 14.27	0.258	0.796 ^†^
**Gender**			0.028	0.867 ^†^
Female	28 (57.1)	21 (42.9)		
Male	35 (55.6)	28 (44.4)		
**WBC**	14.99 ± 8.9	16.75 ± 9.56	1.132	0.258 ^†^
**NEU**	12.62 ± 8.48	16.44 ± 13.72	1.830	0.067 ^†^
**LYM**	1.22 ± 0.81	0.9 ± 0.62	2.499	**0.012 ** ^†^
**MONO**	0.93 ± 0.55	1.03 ± 0.81	0.114	0.909 ^†^
**HGB**	11.9 ± 11.02	14.99 ± 21.29	1.079	0.280 ^†^
**HCT**	31.84 ± 5	31.97 ± 6.25	0.719	0.472 ^†^
**PLT**	302.51 ± 192.9	211.45 ± 136.99	2.871	**0.004 ** ^†^
**PCT**	0.31 ± 0.18	0.23 ± 0.14	2.431	**0.015 ** ^†^
**RDW**	17.08 ± 2.83	18.71 ± 3.28	2.708	**0.007 ** ^†^
**BUN**	32.02 ± 21.18	57.86 ± 31.96	4.673	**0.001 ** ^†^
**CRE**	1.35 ± 0.75	2.03 ± 1.48	2.845	**0.004 ** ^†^
**AST**	58.08 ± 105.08	127.84 ± 161.52	3.002	**0.003 ** ^†^
**ALT**	40.98 ± 120.57	60.27 ± 92.73	2.112	**0.035 ** ^†^
**ALB**	25.46 ± 5.91	23.68 ± 5.49	1.657	0.097 ^†^
**T. BIL**	2.48 ± 3.94	5.05 ± 5.38	3.156	**0.002 ** ^†^
**D. BIL**	1.37 ± 2.89	3.08 ± 3.92	2.794	**0.005 ** ^†^
**CRP**	126.38 ± 98.3	174.07 ± 113.02	2.469	**0.014 ** ^†^
**Emergency Department Outcome**			15.092	**0.001 ** ^†^
Ward	41 (69.5)	18 (30.5)		
ICU	14 (31.1)	31 (68.9)		
**RDW/ALB**	0.71 ± 0.24	0.84 ± 0.95	2.401	**0.016 ** ^†^
**BUN/ALB**	1.32 ± 0.95	2.51 ± 1.44	4.550	**0.001 ** ^†^
**NLR**	13.22 ± 9.76	40.95 ± 99.89	9.650	**0.030**

Given as *mean ± standard deviation.* ^†^: Mann–Whitney U test, statistically significant *p*-values are bolded.

**Table 4:** Cut-off scores, AUC value, sensitivity, specificity, and statistical significance of NLR, RDW/ALB, and BUN/ALB variables according to in-hospital outcomes.

Test Result Variables	*Cutoff*	AUC	Std. Error	*p*	Asymptotic 95% Confidence Interval		*Sensitivity*	*Specificity*
					Lower Bound	Upper Bound		
**RDW/ALB**	>0.83	0.638	0.055	**0.012**	0.538	0.730	48.89	79.66
**BUN/ALB**	>1.86	0.761	0.048	**0.001**	0.668	0.839	64.44	79.66
**NLR**	>13.13	0.658	0.054	**0.006**	0.552	0.764	62.20	51.59

*AUC*: area under the curve.

## Data Availability

The original contributions presented in the study are included in the article; further inquiries can be directed to the corresponding author.

## Abbreviations

The following abbreviations are used in this manuscript:

SBP	Spontaneous bacterial peritonitis
BAR	Blood urea nitrogen/albumin ratio
RAR	Red cell distribution width/albumin ratio
NLR	Neutrophil-to-lymphocyte ratio
ICU	Intensive care unit
ROC	Receiver operating characteristics
RDW	Red blood cell distribution width
PMN	Polymorphonuclear
AUC	Area under the curve
